# Serum Mannan-Binding Lectin in Egyptian Patients With Chronic Hepatitis C: Its Relation to Disease Progression and Response to Treatment

**DOI:** 10.5812/hepatmon.704

**Published:** 2012-04-30

**Authors:** Serag Esmat, Dalia Omran, Gihan A. Sleem, Laila Rashed

**Affiliations:** 1 Department of Internal Medicine, Faculty of Medicine, Cairo University, Cairo, Egypt; 2 Department of Tropical Medicine, Faculty of Medicine, Cairo University, Cairo, Egypt; 3 Department of Biochemistry, Faculty of Medicine, Cairo University, Cairo, Egypt

**Keywords:** Hepatitis C, Interferon, Mannan, Egypt

## Abstract

**Background:**

Chronic hepatitis C virus (HCV) infection is a major worldwide public health problem. Egypt has the highest prevalence of adult HCV infection in the world, averaging 15%–25% in rural communities. Mannan-binding lectin (MBL) is a liver-derived pluripotent serum lectin that plays a role in the innate immune system of the host. It is an acute-phase protein that is involved in the activation of the classical complement pathway. MBL may play a defensive role in HCV infection.

**Objectives:**

To investigate the relationship between MBL concentration and HCV infection in Egyptian patients suffering chronic hepatitis C.

**Patients and Methods:**

Serum samples obtained from 35 Egyptian hepatitis C patients and 30 normal controls were assayed for MBL. MBL concentrations were correlated to disease characteristics and treatment response.

**Results:**

Serum MBL was significantly higher in HCV patients than in controls, but no relationship was found between MBL concentration and disease progression in terms of hepatic fibrosis and inflammation. Responders to interferon (INF)-based therapy had significantly higher serum MBL than non-responders.

**Conclusions:**

We found no association between serum MBL concentration and progression of HCV related liver disease. Responders to INF-based therapy had significantly higher serum MBL than non-responders.

## 1. Background

Mannan-binding lectin (MBL) is an acute-phase protein that is involved in the activation of the classical complement pathway [1]. MBL acts as a direct opsonin by binding to collectin receptors through its collagen domain [2]. Serum levels of MBL in humans are highly influenced by inherited haplotypes that differ at a series of allelic dimorphisms. These differences can influence the structural gene and its promoter region [3][4][5]. Other factors can affect plasma MBL, including growth hormone [6]. The MBL genotype of a person provides only a general idea of the expected plasma concentration and different combinations of haplotypes are associated with a wide range of MBL concentrations [4][5]. Genotypes are good indicators of the average MBL concentrations of populations; however, they provide a less reliable prediction of plasma MBL for individuals [4]. Approximately 60–85% of those infected with HCV cannot eradicate the virus and progress to chronic hepatitis [7][8]. This high rate of viral chronicity may be explained by a failure in the host immune response or by the ability of HCV to defeat host defense mechanisms [7][8]. MBL acts as a recognition molecule for pathogen-associated molecular patterns (PAMPs) which play a role in the initiation and regulation of the immune response [9]. The role of MBL in HCV pathogenesis has received a lot of attention in the last few years [10]. MBL activates the complement system, which results in destruction of pathogens by the membrane attack complex or by complement-mediated phagocytosis. MBL can engage in phagocytosis of infecting agents to modulate the release of proinflammatory cytokines [10][11][12]. Many previous studies of MBL and hepatitis C based diagnoses solely on serology, but included patients with different clinical forms of HCV infection ranging from those with no liver involvement, to those with cirrhosis. This compromises the analyses of the severity of liver involvement [13][14][15][16][17]. Studying the results of liver biopsies is the gold standard for grading hepatitis C. MBL may play an immunomodulatory role during treatment with IFN, as MBL regulates the release of different cytokines from immune cells in response to infection [18].

Hepatitis C-infected patients with MBL haplotypes that were known to confer low MBL concentrations are significantly less likely to respond to interferon (INF) therapy than similar patients who are genetically able to produce greater amounts of MBL [14]. This may be important, because the expense, adverse side-effects, and long duration of antiviral therapy make screening for potential non-responders desirable [14]. The clear implication that MBL plays a major role in elimination of the virus raises the possibility that MBL replacement therapy may be beneficial for hepatitis C carriers with low levels of MBL [14]. However, at least one other study [19] found that susceptibility to hepatitis C infection is not increased by low circulating MBL, and MBL concentration does not play a big role in the course of the disease or patient response to antiviral therapy. If this is the case, MBL replacement therapy would not be beneficial for chronic hepatitis C patients who failed to respond fully to treatment with interferon and ribavirin [19].

## 2. Objectives

We therefore aimed to investigate the relationship between MBL concentration and hepatitis C infection at the protein level in Egyptian patients suffering from chronic hepatitis C.

## 3. Patients and Methods

### 3.1. Patients

Thirty-five randomly selected HCV patients that were candidates for IFN-based therapy and 30 healthy volunteers participated in the study. Patients were recruited from the outpatient clinics of the internal medicine and tropical medicine departments, Kasr Al Aini hospital, Cairo University.

### 3.2. Methods

We collected serum samples from all patients and controls. Serum was tested for HCV antibodies and MBL concentration. Other laboratory tests included fasting blood glucose, aspartate aminotransferase (AST), alanine aminotransferase (ALT), alkaline phosphatase, serum creatinine, serum albumin and complete blood count. Samples were drawn after an overnight fast and were measured using standard commercial methods on a parallel multi-channel analyzer (Hitachi 7170A, Tokyo, Japan). All HCV patients were subjected to the following: 1). quantitative polymerase chain reaction (PCR) assay for HCV before starting treatment and 12 weeks after beginning treatment. Patients that responded to treatment were tested again 6 months after beginning and at the end of treatment. 2) Liver biopsy as a routine measure before starting treatment. 3) Alpha-fetoprotein (AFP). PCR for HCV RNA was performed on samples from controls as well as patients. All participants in the control group tested negative.

HCV RNA was extracted from a 200 μL serum sample using Ana-gen RNA extraction kit (Qiagene, USA) according to the manufacturer’s instructions. cDNA was prepared by Reverse transcription PCR using M-MLV reverse transcriptase (Fermentas, USA). The amplified cDNA was further subjected to 2 rounds of PCR amplifications using a highly conserved primer sequence, upstream primer 5′-GCAGAAAGCGTCTAGCCATGGCGT and downstream primer, 5′-CTCGCAAGCACCCTATCAGGCAGT. The conditions for the first round of PCR were as follows: an initial denaturation step at 95°C for 2 min followed by 30 cycles of 45 s at 94°C, 45 s at 54°C, and 1 min at 72°C. PCR cycling conditions for the second round are as for first round amplification except for an annealing temperature of 54°C.All PCR products (first and second rounds) were analyzed on 1.8% agarose gel prepared in 0.5% TBE buffer, stained with ethedium bromide and visualized by ultraviolet [20]. Ultrasound-guided biopsy was performed for all patients using 18–20 gauge needles (, GHATWARY MEDICAL SUPPLIES, Alexandria, Egypt). Diagnostic liver biopsy specimens were scored using the Ishak modified histological activity index (HAI) and Ishak fibrosis stages [21]. Before treatment, MBL was measured in serum samples using an enzyme-linked immunosorbent assay (ELISA) kit (Hycult Biotech, Uden, Netherlands). Briefly, a standard curve was prepared by serial dilution of standards supplied with the assay. Standards and serum samples were added to a 96-well plate containing immobilized antibodies specific to MBL. The plate was incubated at room temperature for 2 h, with gentle shaking. Following extensive washing, bound MBL was detected using an HRP-conjugated polyclonal antibody against the target protein. The detection antibody was incubated for 2 h at room temperature, and detected using 3,5,3′,5′-tetramethylbenzidine (TMB) and 3% H2O2. The reaction was terminated by the addition of sulfuric acid provided with the kit. Optical density was detected using a microplate reader at 450 nm with a wavelength correction reading at 540 nm. Concentrations of unknowns were calculated from the standard curve [22].

Patients were given a course of antiviral therapy consisting of Peginterferon alfa-2a (Pegasys®) 180 mcg (regardless of body weight) subcutaneously once weekly and 10.6 mg/kg ribavirin orally every day (combination therapy). After 12 weeks of treatment, patients with a reduction of more than 2 logs in PCR results were deemed to be responders and continued treatment for a total of 48 weeks. Responders (n = 20) were tested again 6 months after the end of therapy using PCR to ensure sustained virological response (SVR). Patients with PCR scores that were not at least 2 logs lower after 12 weeks of therapy than at baseline were deemed to be non-responders (n = 11). Four patients were lost to follow-up during INF-based therapy. Informed consent was obtained from all patients and controls.

### 3.3. Statistical Analysis

All patient data were tabulated using Excel 7. Data were then processed using SPSS (Statistical Package for Science and Society) version 17.0 (SPSS Inc., Chicago, IL, USA) for Windows 7 (Microsoft, Corporation, NY, USA). Descriptive statistics are presented as means ± standard deviations (SD) for quantitative variables. All qualitative data are expressed as frequencies (number) and percentages. Groups were compared using chi-square test and Fisher’s test, as appropriate for qualitative data. Independent sample t-test was used for comparing normally distributed quantitative variables between groups and non-parametric Mann–Whitney test and Kruskal–Wallis test were used to compare abnormally distributed quantitative variables between groups. Correlation analysis was used when appropriate. P values lower than 0.05 were considered statistically significant for all tests.

## 4. Results

**Figure 1:**
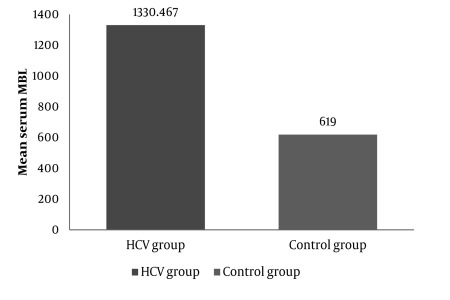
Mean Serum MBL Concentration Was Significantly Higher in Chronic Hepatitis C Patients Than in Controls (P < 0.001; Mann–Whitney Test).

**Figure 2:**
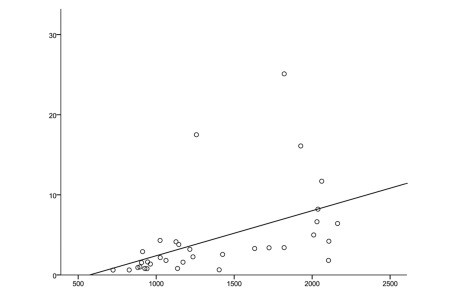
Correlation Between Serum MBL Level and Alpha-Fetoprotein (AFP) in the HCV Infected Group (R = 0.59, P < 0.001; Spearman Correlation Test).

Our study included 35 patients between 19 and 56 years of age (20 males, 15 females, mean age: 37.43 ± 9.65). Baseline characteristics of patients are summarized in Table 1. The control group included 30 healthy subjects between 18 and 55 years of age (18 males, 12 females, mean age: 36.8 ± 11.885). The characteristics of the control group are shown in Table 1. We performed multivariate analysis to adjust for age and gender effects, but neither age nor gender significantly affected other variables (P ˃ 0.05). MBL concentrations were significantly higher in chronic hepatitis C patients (P ˂ 0.001) than in controls (1330.47 ± 497.81 ng/mL in patients vs. 619 ± 136 ng/mL in the controls; Figure 1). There was a significant positive correlation between serum MBL level and alpha-fetoprotein in the HCV infected group (Figure 2). Within the HCV group, the fibrosis scores were 1, 2, and 3 in 16, 12, and 7 patients, respectively. The activity score for inflammation was 1, 2, and 3 in 10, 7, and 18 patients, respectively (Table 2). MBL concentrations did not differ significantly between different grades of liver fibrosis and liver inflammation (Table 2). Twenty patients showed SVR to INF-based therapy, while 11 patients were non-responders. Serum MBL was significantly higher in responders than in non-responders (Table 3).

**Table 1:** Baseline Characteristics of HCV Patients and Healthy Controls

	** Patients**			** Control Group**		
	**Minimum**	**Maximum**	**mean ± SD**	**Minimum**	**Maximum**	**mean ± SD**
Age, y	19	56	37.43 ± 9.65	18	55	36.80 ± 11.89
Body weight, Kg	53	105	79.79 ± 14.61	66	107	89.43 ± 10.85
Blood glucose, mg/dL	60	321	103.50 ± 43.38	68	112	87.60 ± 12.36
Creatinine, mg/dL	0.44	1.30	0.82 ± 0.20	0.4	1.1	0.74 ± 0.18
AlP a, IU/L	6.20	252.00	79.09 ± 41.45	56	129	81.53 ± 20.53
AST a, IU/L	10	283	54.50 ± 49.76	13	30	18.93 ± 5.66
ALT a, IU/L	8	243	62.51 ± 46.18	12	30	17.43 ± 5.28
T. Bilirubin a, mg/dL	0.24	1.80	0.83 ± 0.34	0.22	1	0.52 ± 0.21
Albumin, gm/dL	3.2	5.10	4.68 ± 0.99	3.9	4.9	4.41 ± 0.30
Hb a, gm/dL	11.0	16.9	14.11 ± 1.54	10.9	14.8	12.65 ± 0.82
Platelet/cmm	110,000	347000	203471 ± 58514	222.000	390000	296533.30 ± 48131.09
WBCs a /cmm	3100	11720	6775.14 ± 1895.65	4200	9000	6017.66 ± 1421.04
AFP a, ng/mL	1.6	44.1	5.29 ± 8.19	--	--	--
MBL a, ng/mL	146.3	2163.0	1330.47 ± 497.81	502	916	619 ± 136

^a^ Abbreviations: AFP, alpha-fetoprotein; ALP, alkaline phosphatase; ALT, alanine aminotransferase; AST, aspartate aminotransferase; Hb, hemoglobin; MBL, mannan binding lectin; T Bilirubin, total bilirubin; WBC, white blood cell

**Table 2:** Serum MBL in Different Grades of Liver Fibrosis and Hepatic Inflammation in the Patients Infected With HCV

	**Frequency, No.**	**MBL a, ng/mL, mean ± SD**	**P value**
Fibrosis score	35		0.79 b
1	16	1285.46 ± 438.42	
2	12	1317.02 ± 540.5	
3	7	1447.9 ± 513.6	
Activity score	35		0.52 b
1	10	1259.82 ± 622.11	
2	17	1337.5 ± 458.5	
3	8	1471.3 ± 480.8	

^a^ Abbreviation: MBL, Manran Binding Lection

^b^ Kruskal–Wallis test was performed to obtain the P value

**Table 3:** Serum MBL Among Responders and Non-Responders to INFBased Therapy a

**Patient Groups (n = 35)**	**No.**	**MBL, ng/mL**
Non responders b	11	1023.78 ± 163.11
Responders c	20	1882.36 ± 291.34
Missed d	4	1879.87 ± 237.25

^a^ P ˂ 0.001; Kruskal–Wallis test was performed to obtain the P value

^b^ Less than 2 logs reduction in PCR after 12 weeks of therapy

^c^ More than 2 logs reduction in PCR after 12 weeks of therapy and negative PCR at week 72 (SVR)

^d^ More than 2 logs reduction in the PCR after 12 weeks of therapy, but dropped out of study before completion of the 48-week course of therapy

## 5. Discussion

Egypt has the highest prevalence of adult HCV infection in the world, as 15%–25% of the population in rural communities is infected [23][24]. The outcome of HCV infections are usually associated with the interaction between the virus and the host immune response [25]. Mannan-binding lectin (MBL) is a liver-derived pluripotent serum lectin that plays a role in the host immune system [12]. The current study investigated the serum MBL level among Egyptian patients with chronic hepatitis C. We found that chronic HCV patients had higher MBL concentrations than healthy controls. This can be explained by the fact that MBL is an acute phase reactant [26]. However, our results contradict those of Yuen M-F et al. 1999 [27] who reported that asymptomatic HCV patients had lower MBL levels than controls. While not statistically significant, MBL concentration tended to increase with the grades of fibrosis and hepatic inflammation. Other authors have also observed a statistically non-significant link between high serum MBL and more severe fibrosis [28]. Further studies with larger numbers of patients are needed to assess this relationship. Although the association between the concentration of MBL and hepatitis severity was not statistically significant, it may still be real, and may reflect MBL’s role as an acute phase reactant. This is consistent with the highly significant increase in MBL in hepatitis C patients as compared to normal controls that we observed.

Serial AFP measurements in patients with chronic hepatitis C are useful for identifying persons with advanced fibrosis and help to determine which patients need periodic liver ultrasound screening to detect HCC [29]. In our study, a significant positive correlation was found between serum MBL and AFP level. Moreover, both increased as the fibrosis stage became more advanced. This finding supports the hypothesis that MBL may be related to disease progression. In the current study, non-responders to INF-based therapy had significantly lower MBL concentration than responders. However, both had significantly higher MBL levels than normal controls. The lower levels of MBL in non-responders may be explained by the inhibitory effect of HCV on MBL production from the liver [13]. Those with higher levels of MBL may have a better ability to eliminate the virus [14]. MBL may play an immunomodulatory role during treatment with IFN, as MBL regulates the release of various cytokines from immune cells in response to infection [18].

Progression of chronic hepatitis B and C are reported to be associated with MBL insufficiency [13][14][15][30]. Thio et al. [30] reported that recovery from hepatitis B infection was associated with high MBL serum levels while persistence of the virus was associated with low MBL serum levels. Liver cirrhosis has also been linked to low serum levels of MBL [27]. Additionally, spontaneous bacterial peritonitis (SBP) is much more common in MBL-deficient cirrhotics [27]. SBP is a serious disease and prevention of SBP in MBL-deficient cirrhotics with MBL therapy would be a valuable advance. This disease is a good candidate for a clinical trial of MBL therapy. Responders to INF-based therapy had significantly higher serum MBL than non-responders. MBL may be a predictor of antiviral therapy response in HCV patients. Further studies are needed to evaluate MBL replacement therapy, which may improve the response to antiviral therapy in these patients. The possible relationship between serum MBL concentration and progression of HCV-related liver disease deserves further evaluation with a larger number of patients.
